# Clinical malaria diagnosis: rule-based classification statistical prototype

**DOI:** 10.1186/s40064-016-2628-0

**Published:** 2016-06-30

**Authors:** Francis Bbosa, Ronald Wesonga, Peter Jehopio

**Affiliations:** School of Statistics and Planning, Makerere University, P.O. Box 7062, Kampala, Uganda; East African Statistics Institute, P.O. Box 11140, Kampala, Uganda

**Keywords:** Statistics, Malaria diagnosis, Rule-based classification, Sensitivity, Specificity

## Abstract

In this study, we identified predictors of malaria, developed data mining, statistically enhanced rule-based classification to diagnose malaria and developed an automated system to incorporate the rules and statistical models. The aim of the study was to develop a statistical prototype to perform clinical diagnosis of malaria given its adverse effects on the overall healthcare, yet its treatment remains very expensive for the majority of the patients to afford. Model validation was performed using records from two hospitals (training and predictive datasets) to evaluate system sensitivity, specificity and accuracy. The overall sensitivity of the rule-based classification obtained from the predictive dataset was 70 % [68–74; 95 % CI] with a specificity of 58 % [54–66; 95 % CI]. The values for both sensitivity and specificity varied by age, generally showing better performance for the data mining classification rules for the adult patients. In summary, the proposed system of data mining classification rules provides better performance for persons aged at least 18 years. However, with further modelling, this system of classification rules can provide better sensitivity, specificity and accuracy levels. In conclusion, using the system provides a preliminary test before confirmatory diagnosis is conducted in laboratories.

## Background

Clinical diagnosis is the most widely applied and reliable method for drawing conclusions about the patient’s malarial status (Blobel et al. 2013). It is often the only feasible approach in many circumstances, such as in rural and areas of high prevalence where patients may be willing to incur treatment costs, but are unable to pay for charges related to diagnosis (Danyliv et al. 2013; Hypponen et al. 2013; Raknes et al. 2013). This approach is inexpensive to perform and requires no specialized equipment (Epstein and Blumenfield 2001). In Uganda, a number of ongoing computer based initiatives aimed at strengthening existing health care systems at the Health facility levels have been undertaken in recent years (Wesonga et al. 2015). These initiatives include systems such as the Code 8 (Fallon 2013) and other UNICEF initiatives such as MobileVRS, DevTrac, mHealth, mTRAC, HMIS. However, all these systems are basically disease surveillance innovations and hence still lacking as far as online clinical based medical diagnosis and inference is concerned. Save for the epidemiological statistics and stocktaking, the current systems lack statistical modules such as predictive models and goodness of fit to generate necessary and timely statistics to address the knowledge gaps and challenges in the existing systems. Above all, malaria is dynamic with symptoms varying from one patient to another and can be sometimes difficult to distinguish from other diseases including typhoid and hypertension (Guwatudde et al. 2015).

Data mining brings a set of tools and techniques that can be applied to these data so as to discover hidden patterns that provide health care professionals an additional source of knowledge for making decisions (Prasanna 2011). Accordingly, (Baylis 1999; Hardin and Chhieng 2007; Hypponen et al. 2013) concur that health care is a very large domain with enormous opportunities for data mining. Data mining brings the facility to discover patterns and correlations that could be hidden within the data repository thus, the expert knowledge of the health professionals is enhanced to uncover relevant patterns; and to be more empowered since decisions rest with healthcare professionals, but not the information system experts (Baylis 1999).

According to (Epstein and Blumenfield 2001), data miners choose the data mining technique by using two main parameters; the main goal of the problem to be solved and the structure of the available data. Rule-based classifiers are explored in this study to provide a set of classification rules that can be used later to evaluate a new case and classify an already predefined set of classes for malaria. This technique is used in this study to provide a precursor to medical diagnosis of malaria using testing tools such as sensitivity and specificity of system’s malarial diagnosis. Based on patients’ profile, history, physical examination, diagnosis and utilizing previous treatment patterns, new treatment plans can be effectively recommended (Cunha et al. 2013; Prasanna 2011).

With the third highest number of deaths in Africa (Nankabirwa et al. 2009; Tangpukdee et al. 2009), malaria is the leading cause of morbidity in Uganda and is responsible for up to 40 percent of all outpatient visits, 25 percent of all hospital admissions and 14 percent of all hospital deaths. It was established that 95 percent of the population is at risk (Okello et al. 2006), as malaria kills between 70,000 and 100,000 children every year, a death toll that far exceeds that due to HIV/AIDS. According to many studies including (Yeka et al. 2012), malaria was an obstacle to achieving some of the most vital Millennium Development Goals (MDGs). Since 1998, Uganda has moved to adopt several key regional and international goals and targets including the overall Roll Back Malaria (RBM) Goals, the MDGs and the Africa-specific Abuja summit declaration; and all these called for the reduction of morbidity and mortality due to malaria through the scaling up of key malaria interventions to at least 60 percent of the risk populations.

Uganda attempted to meet MDG targets for the Universal Primary Education, but was not able to meet targets for the sixth goal that focused on malaria (Korenromp et al. 2013; Nankabirwa et al. 2009). The fact that Uganda failed to meet MDG 6c that is, “Have halted by 2015 and begun to reverse the incidence of malaria and other major diseases” was among the motivating factors for this study. In a 2007 resolution, the World Health Assembly called for a 75 % reduction in malaria case incidence rates by 2015 compared to levels in the year 2000 (Kyabayinze et al. 2010). The increase in mortality and morbidity due to malaria globally and specifically in Uganda could be possibly due to high malaria diagnosis related user fees such as doctors’ consultation fees, costs incurred on moving to a health facility and laboratory costs. Other challenges include; the amount of time a patient spends in queuing at the Hospital just to see a doctor, time spent waiting for laboratory results and duration of transit from home to hospital. In addition, other factors such as bad weather, poor road network, lack of transport means and lack of money at that time might also contribute to the increase in malaria mortality rates (Probst et al. 2007; Tediosi et al. 2008). These barriers to providing a timely diagnosis for malaria could be addressed by providing an online malarial screening system that provides the patient with information regarding his or her likely diagnosis, before he receives a confirmatory diagnosis.

In many developing countries, malaria is diagnosed via either the microscopic examination of blood films or a rapid diagnostic test (Kyabayinze et al. 2008; Piola et al. 2005). It is reported that on average, the microscopic diagnosis usually takes about 30 min and requires a laboratory technologist. Alternatively, a rapid diagnostic test takes on average 15 min to get the results can be used anywhere without a qualified Microscopist (Fallon 2013; Seidel et al. 2006). This study explored data mining techniques with statistical modules such as predictive models and goodness of fit that could generate necessary and timely statistics to address the knowledge gaps and challenges in the existing systems.

## Methods and data sources

The study used malaria specific reviewed secondary data that we extracted from Form V (Patient Record Form) of the Ministry of Health from Kalisizo Hospital, a public Hospital in Rakai district in Southern Uganda and Kisubi Hospital, a private Hospital in Wakiso district in Central Uganda. The patient extraction process from Form V involved identification, recording and cleaning of the data in an Excel sheet. These data were used to develop, test and validate the malaria rules. Further, the study assessed differences in the predictors captured by public and private hospitals respectively, as well as any limitations arising from management of patients due to type of ownership of the hospital.

### Study population and distribution

This study targeted persons who were 5 years and above, presenting to the hospital with malaria like symptoms. Persons below 5 years were not included due to their inability to explicitly express their symptoms to the medical worker. The variables comprised of ten signs and symptoms including; Fever, Splenomegaly, Jaundice, Joint pains, Fits, Rigors, dark urine, vomiting, febricity, drowsiness, plus malaria test outcome and the patient’s age (Probst et al. 2007; Tediosi et al. 2008).

Table 1 shows the distribution of the sample and prevalence of malaria. A total of 973 records were extracted from the records showing a relatively higher prevalence of malaria (76.2 %) in Kisubi Hospital than in Kalisizo Hospital (12.3 %). The highest proportion of patients was those aged 18 and above (44.4 %), followed by the 5 to 9 year olds (38.2 %) and then 10 to 17 year olds (17.4 %). The difference in the malaria prevalence by hospital could be explained by susceptibilities of their locations to the anopheles mosquitoes. Whereas Kisubi Hospital is located at the shores of lake victoria, showing higher vulnerability of getting infected with malaria, Kalisizo Hospital’s location is not in such an environment that exposes its inhabitants to many risks of malarial infection. The other factor could be that, whereas Kisubi Hospital serves a number of communities including; the local community, two primary schools, three secondary schools, two tertiary schools and one university; Kalisizo Hospital serves only the local community around it.

**Table 1:** Malaria prevalence, signs and symptoms by hospital

Malaria	Kalisizo Hospital	Kisubi Hospital	Overall
	Percentage (%)	Percentage (%)	Percentage (%)
Proportion with malaria	12.3	76.2	22.2
*Signs and symptoms*			
Febrile	98.2	53.0	91.2
Fever	79.7	20.5	70.5
Rigors	94.8	78.8	92.3
Drowsy	94.3	57.0	88.5
Fits	99.9	91.4	98.6
Dark urine	98.9	97.4	98.7
Joint pains	93.2	90.7	92.8
Vomit	96.4	63.6	91.3
Jaundice	100.0	91.4	98.7
Splenomegaly	97.7	73.5	93.9
Total	822	151	973

Table 2 further shows malaria signs and symptoms by diagnosis. The analysis shows higher proportions of fits, dark urine, joint pains and jaundice as signs and symptoms that have strong relationships with a positive malarial outcome.

**Table 2:** Malaria signs and symptoms by diagnosis

Signs and symptoms	Malaria diagnosis		
	Negative percentage (%)	Positive percentage (%)	Total percentage (%)
Febrile	97.5	69.0	91.2
Fever	78.2	43.5	70.5
Rigors	94.9	83.3	92.3
Drowsy	93.7	70.4	88.5
Fits	99.7	94.4	98.6
Dark urine	98.7	98.6	98.7
Joint Pains	92.9	92.6	92.8
Vomit	95.2	77.3	91.3
Jaundice	99.7	94.9	98.7
Splenomegaly	97.5	81.5	93.9
Total	757	216	973

### Data analysis

The classical scheme of knowledge discovery (Qin et al. 2009; Wu et al. 2008) in form of rule-based classifiers was applied to patients’ records collected from two hospitals. A set of classification rules were developed, evaluated for their reliability to diagnose malaria on a new instance using a predetermined set of rules. Rule-based decision methods grouped factors providing an explicit knowledge model, which could be expressed by formal rules so as to be applied for further prediction. The technique consisted of a list of rules of the form “if A and B and C, then class X”, where rules for each response variable (class) were grouped together. A Positive malaria result was classified by finding the first rule whose conditions were satisfied by the case; if no rule was satisfied, the case was assigned to a default class (Zurovac et al. 2008). The process of knowledge discovery in databases (KDD) in malaria diagnosis comprised of many phases namely data cleaning, data integration, data selection, data transformation, data mining, pattern evaluation and knowledge representation (Beniwal and Arora 2012).

Before data are imported into the system, it is advisable that data processing is mandatorily carried out (Beniwal and Arora 2012) through data cleaning and multiple imputation techniques (Wesonga 2015). In this study, only 2 % (about 20 values) of the data were missing on age; further consultations and review of the Form V identified 18 of the values that were missing completely at random while the remaining 2 values for age were identified from a subsidiary source with the doctor’s guidance. Except for age, numeric attributes were normalized to [0, 1] for example, 0 for normal (37 °C) and 1 for temperatures higher than normal respectively. Data were then exported as comma-separated values (CSV) into the malaria system prototype.

### Interpretation of the algorithm for clinical malaria diagnosis

The study used the Information Gain (ID3) algorithm to select the attribute that minimized the value of entropy and hence maximizing the information gain. Entropy is an information-theoretic measure of the ‘uncertainty’ contained in a training set due to the presence of more than one possible classification (Hand et al. 2001).

Given two classe*s* (P and N), assuming that there were p instances of class P and n instances of class N, then the amount of information (in bits) that is needed to decide if an observation in the training data belonged to P or N is defined by;

$$I\left( {p,n} \right) = - \left( {\frac{p}{p + n}} \right)log_{2} \left( {\frac{p}{p + n}} \right) - \left( {\frac{n}{p + n}} \right)log_{2} \left( {\frac{n}{p + n}} \right)$$

Thus, assuming attribute fever, the entropy, which is the expected information needed to classify objects in all classifiers/rules is given as;

$$E\left( {Fever} \right) = \sum\limits_{i = 1}^{2} {\left( {p_{i} + n_{i} } \right)/\left( {p + n} \right)I\left( {p_{i} ,n_{i} } \right)}$$

The subscript *i* refers to the number of classes for the attribute. Therefore, the encoded information gained by branching on Fever is defined as $Gain\left( {Fever} \right) = I\left( {p, n} \right) - E \left( {Fever} \right)$.

**Fig. 1:**
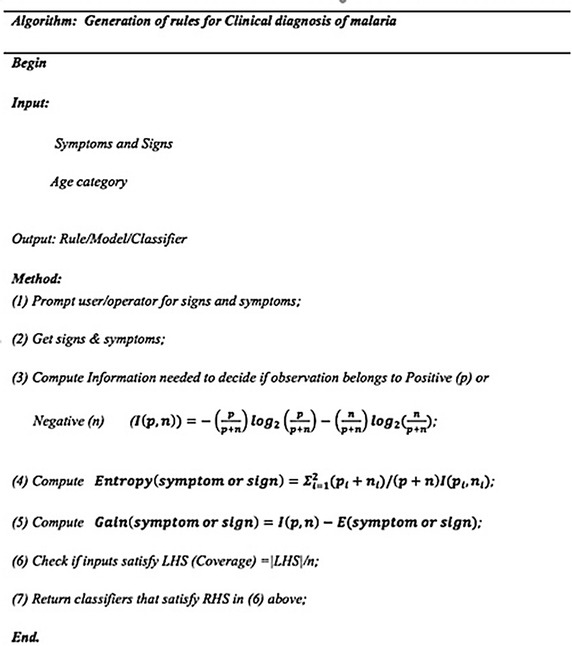
Clinical diagnosis algorithm

The process was repeated for each attribute by iterating through every unused attribute of the set and calculating the entropy of the attribute. The attribute with the smallest entropy (or largest information gain) value could then be selected. The set was then split by the selected attribute to produce subsets of the data. The algorithm continues to recur on each subset, considering only attributes that have not been selected before. Figure 1 shows a summary of the algorithm that generates rules for the clinical diagnosis of malaria.

### Output evaluation

The study applied sensitivity and specificity to evaluate the rules for the clinical malaria diagnosis algorithm. Sensitivity was computed to show the proportion of patients with malaria who tested positive while specificity represented the proportion of the patients who tested negative while applying the data mining classification rules. Other related evaluation approaches included; positive and negative predictive values for the malaria diagnosis classification rules.

Further evaluation of the classification rules for quality were assessed by following the steps outlined (Tan et al. 2002). Comparisons were done for the left-hand side, LHS and right-hand side, RHS of the rule.

- Coverage: Fraction of records that satisfied antecedent of the rule
- Coverage = |LHS|/n
- Accuracy: Fraction of records covered by the rule that belong to class on RHS
- Accuracy = |LHS n RHS|/|LHS|

Hence, accuracy of the rule used is the fraction of instances that satisfied both the antecedent and consequent of a rule, normalized by those that satisfied the antecedent (Qin et al. 2009). It should be noted that not all inputs satisfy the condition because initially, all attributes are possible inputs in the model. However, the process is repeated recursively until we eventually end up with the significant variables that predict malaria cases by age category and thus the coverage (LHS). In other words, we only present the relevant variables in the model.

## Findings of the study

To facilitate the use of the data mining classification rules, an automated system, the clinical malaria diagnosis system was developed. The problem was divided into three components: input, process and output. Outputs are the goal of the solution to the problem. Inputs are the information used to solve the problem. Processes are steps needed to convert input information into desired output results. The study also came up with a tool for collecting the above components as shown in the input process and output (IPO) diagram.

**Fig. 2:**
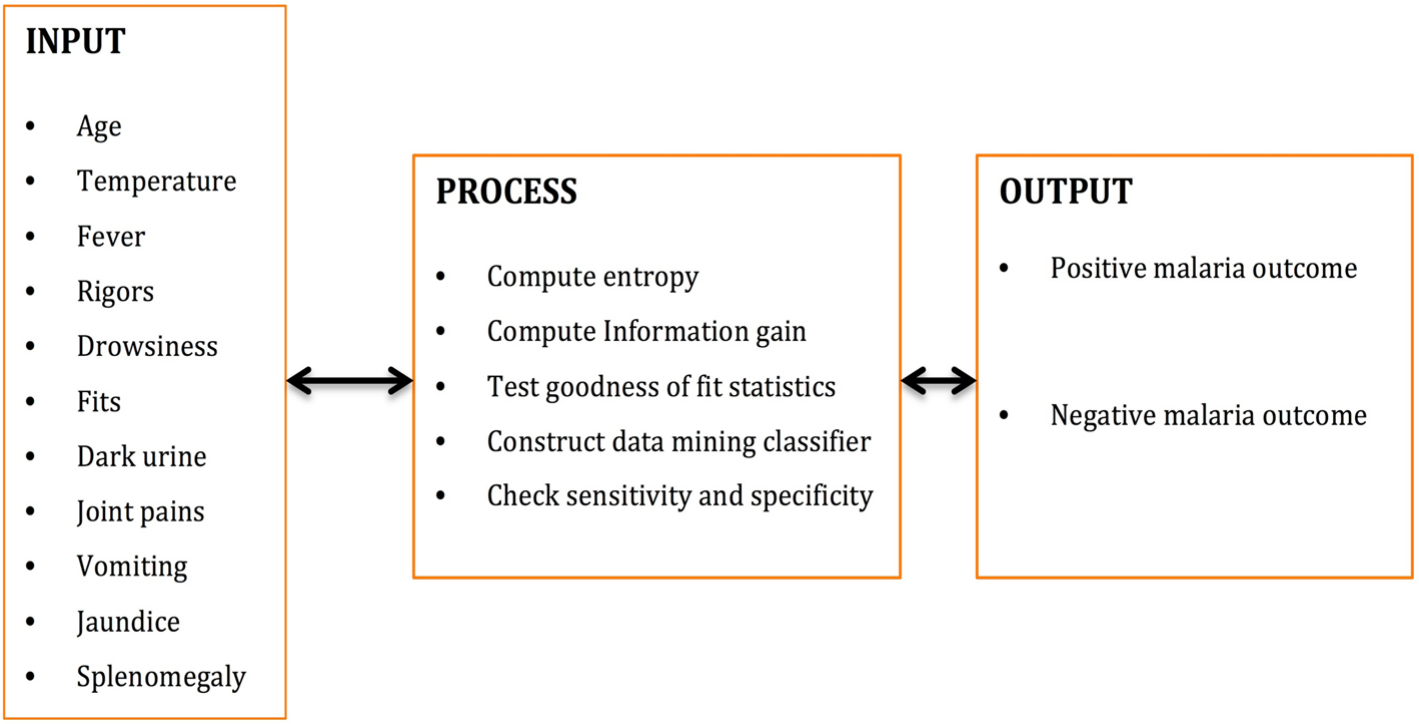
Input output diagnosis for malaria based on signs and symptoms

Figure 2 shows inputs, processes and outputs for the study. It can be noted that inputs included ten signs and symptoms of malaria as well as patients’ age. These inputs undergoes the following processes that is, entry into the system, entropy and information gain computation for each input, rule construction/derivation, sensitivity and specificity of outputs and rule quality evaluation in order to determine the malaria outcome of any person presenting with malaria symptoms and signs.

**Fig. 3:**
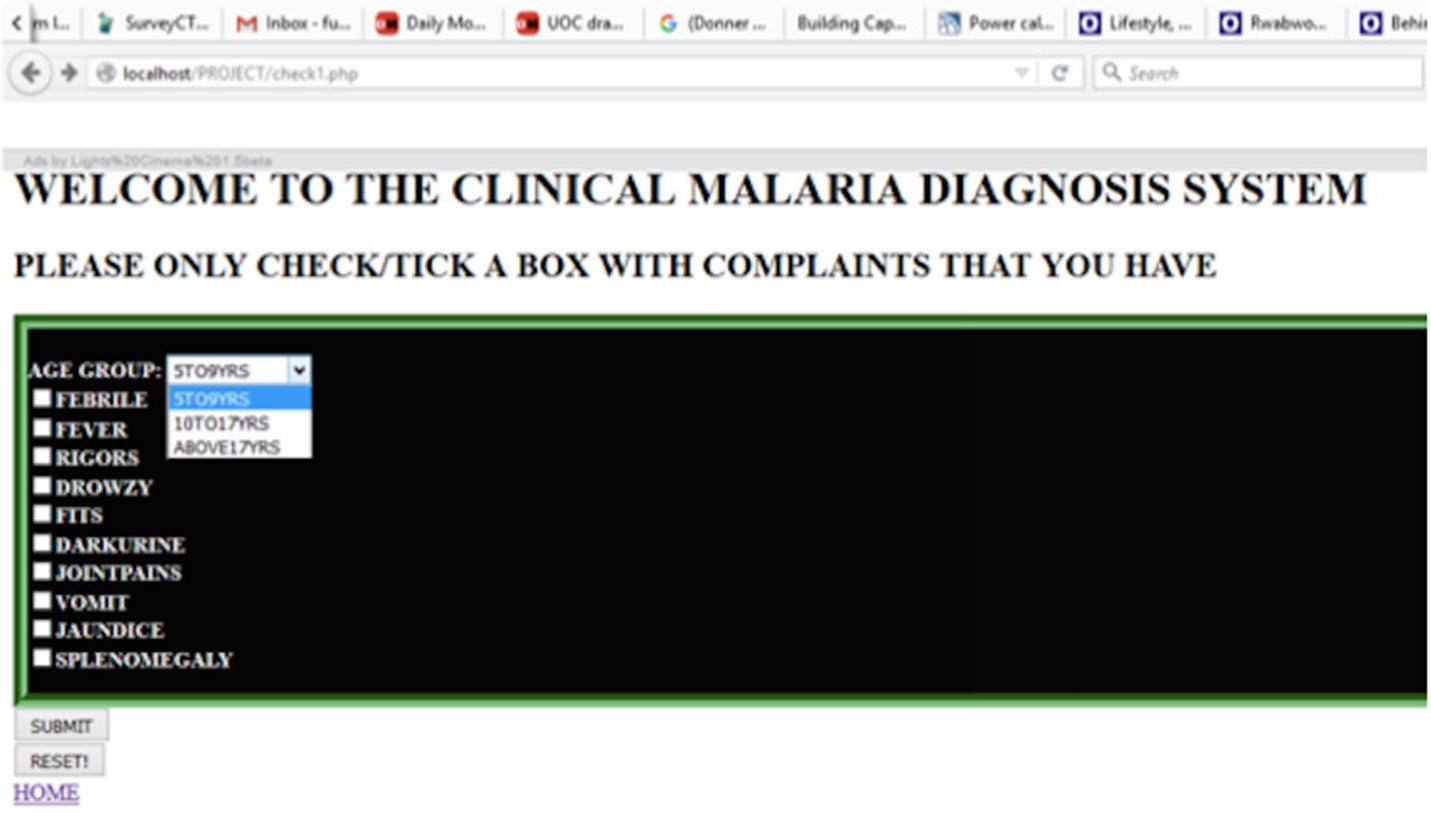
Patient rule-based online interface

Figure 3 shows an online-based malaria rule based interface, where the patient inputs by selecting (checking) on the user system interface all symptoms or signs being presented with; the system processes and outputs an advisory note classifying the patient as having or not having malaria. The design of the online system is based on the algorithm.

### Predictors of clinical malaria

**Fig. 4:**
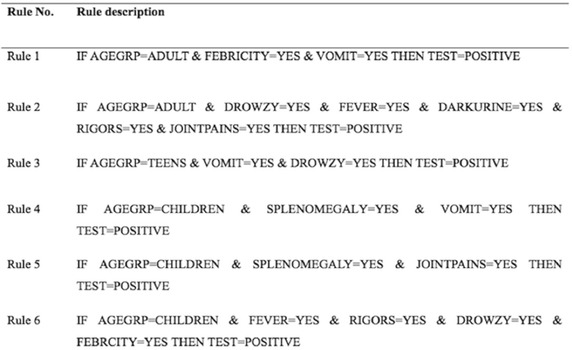
Malaria diagnosis data mining and classification rules

Figure 4 shows malaria outcome based on the prescribed predictors, rules and classifiers for the models of classification given the data mining rules.

Table 3 illustrates data mining rule-based malaria diagnosis and classification against the symptoms and signs that a patient presents with. Rule 1 and 2 were constructed for adults, rule 3 for teenagers and rules 4, 5 and 6 for children aged from 5 to 9 years of age.

**Table 3:** Malaria rule based diagnosis and classification against the symptoms and signs

Symptoms and signs	Age category					
	Adults (18 years and above)		Teens (10–17 years)	Children (5–9 years)		
	Rule 1	Rule 2	Rule 3	Rule 4	Rule 5	Rule 6
Febricity	Yes	–	–	–	–	Yes
Drowsiness	–	Yes	Yes	–	–	Yes
Fever	–	Yes	–	–	–	Yes
Dark urine	–	Yes	–	–	–	–
Rigors	–	Yes	–	–	–	Yes
Joint pains	–	Yes	–	–	Yes	–
Vomiting	Yes	–	Yes	Yes	–	–
Splenomegaly	–	–	–	Yes	Yes	–
Malaria outcome	POS	POS	POS	POS	POS	POS

*POS* positive malaria outcome

The predictors of malaria formed a logical clinical combination of the above attributes.

### Statistical predictive models

The amount of Information that was gained by branching on each of the attributes is illustrated in Table 4.

**Table 4:** Information in binary digits gained by branching on each attribute

Symptom/sign	Information gain
Overall I (p,n)	0.5380
Age group	0.0280
Febricity	0.0040
Fever	0.0010
Rigors	0.0005
Drowzy	0.0020
Darkurine	0.0010
Joint pains	0.0000
Vomit	0.0030
Splenomegaly	0.0130

Hence, the classification rules were formulated by splitting the attributes in the following descending order: Age group, Splenomegaly, Febricity, Vomit, Drowsy, Fever, Dark urine, Rigors, Joint pains respectively. Age group meant that the information gained by branching on attribute Age group is 0.028 BITs and since it had the highest information gain, the algorithm started splitting and selecting it. The above procedure was repeated for the other attributes. The overall information, I(p,n) means the amount of information needed to decide whether a patient was negative or positive given the case study was calculated as 0.538 binary digits (BITs).

### Goodness of fit statistics

To test the goodness of fit of the system output, sensitivity and specificity analyses were conducted. Sensitivity refers to the proportion of patients with malaria who tested positive while specificity is the proportion of the patients who tested negative with the classification rules.

In Table 5, we present the number of patients in the predictive and training data sets. We establish the number of patients with positive and negative clinical malaria diagnosis and those determined using the classification rules. The total number of patients classified in the two hospitals was 973 of which 822 patient records were used as training data for the mining classification rules. Overall, 216 patients were positive of which 101 were from Kalisizo hospital while 115 were from Kisubi hospital. Of these, 757 patients were negative of which 721 were from the training dataset (Kalisizo hospital) while 36 were from the predictive dataset (Kisubi hospital).

**Table 5:** Predicted number of patients using rule-based classification against true clinical diagnosis by age

Predicted malarial status	True clinical diagnosis of malaria							
	5–9 Years		10–17 Years		18 Years and above		Total	
	Positive	Negative	Positive	Negative	Positive	Negative	Positive	Negative
*(a) Predictive dataset: Kalisizo Hospital*								
Positive	6	4	10	3	65	8	81	15
Negative	5	4	4	3	25	14	34	21
Total	11	8	14	6	90	22	115	36
*(b) Training dataset: Kisubi Hospital*								
Positive	56	22	20	19	11	15	90	16
Negative	5	270	5	105	4	290	11	705
Total	61	292	25	124	15	305	101	721

In Table 6, we present the goodness of fit statistics by age category. Given that the rules for malaria diagnosis vary by age category, presentations for the goodness of fit statistics for different age groups and the two hospitals are made. Primarily, records from Kalisizo Hospital were used as a training dataset while records from both Kisubi Hospital were used as the predictive dataset. In data mining, the training dataset is used to develop the rules, while the predictive dataset test the sensitivity and specificity of the rule-based system. However, in our analysis, we present the goodness of fit statistics for both the predictive and training datasets. The goodness of statistics measures includes; sensitivity, specificity, positive predictive value and negative predictive values respectively. The overall, sensitivity of the rule-based classification on the predictive dataset obtained from Kisubi hospital was 70 % [68–74; 95 % CI] with a specificity of 58 % [54–66; 95 % CI]. These values varied by age categories, generally showing better performance with age as shown for sensitivity analysis; 5–9 year olds 55 % [43–66; 95 % CI], 10–17 year olds 71 % [62–80; 95 % CI] and 18 and above year olds 72 % [69–76; 95 % CI]. Generally, the performance of the rule-based classification was found to have a better performance for the older patients than the younger ones.

**Table 6:** Goodness of fit for rule-based classification of patients’ malaria outcome by age

Goodness of fit statistics	5–9 Years		10–17 Years		18 Years and above		Total	
	%	95 % CI	%	95 % CI	%	95 % CI	%	95 % CI
*(a)* *Predictive dataset: Kalisizo Hospital*								
Sensitivity	0.55	[0.43–0.66]	0.71	[0.62–0.80]	0.72	[0.69–0.76]	0.70	[0.68–0.74]
Specificity	0.50	[0.35–0.67]	0.50	[0.36–0.70]	0.64	[0.58–0.73]	0.58	[0.54–0.66]
Positive predictive value	0.60	[0.46–0.77]	0.77	[0.67–0.91]	0.89	[0.87–0.93]	0.84	[0.82–0.89]
Negative predictive value	0.44	[0.30–0.62]	0.43	[0.30–0.63]	0.36	[0.31–0.45]	0.38	[0.34–0.46]
*(b)* *Training dataset: Kisubi Hospital*								
Sensitivity	0.92	[0.90–0.93]	0.80	[0.74–0.83]	0.73	[0.63–0.76]	0.89	[0.87–0.90]
Specificity	0.92	[0.91–0.93]	0.85	[0.79–0.87]	0.95	[0.93–0.96]	0.98	[0.97–0.98]
Positive predictive value	0.72	[0.67–0.74]	0.51	[0.41–0.56]	0.42	[0.30–0.45]	0.85	[0.82–0.86]
Negative predictive value	0.98	[0.98–0.98]	0.95	[0.94–0.96]	0.99	[0.98–0.99]	0.98	[0.98–0.99]

The rule based classification method was used to analyse hospital data in order to identify relevant predictors of clinical malaria. In reference to the stated rules, antecedents and consequents were used to evaluate the quality of each classification rule shown in Table 7. Antecedents are the records that satisfied the left-hand side, LHS (the IF part) of the rule whereas the consequents are the records that satisfied the right-hand side, RHS (the THEN part) of the rule.

**Table 7:** Quality of the classification rule evaluation for Kisubi Hospital (n = 151)

Age group	Adults (18+ years)		Teens (10–17 years)	Children (5–9 years)		
Coverage by age group (%)	74.20		13.30	12.50		
RULE	RULE (1)	RULE (2)	RULE (3)	RULE (4)	RULE (5)	RULE (6)
Coverage (%)	17.90	0.00	15.00	21.10	5.30	0.00
Accuracy (%)	85.00	0.00	66.70	75.00	100.00	0.00
Sample (n)	112		20		19	

The second row of Table 6 shows the percentage composition of each age group in the predictive data. The fourth row shows the percentage of records that satisfied the antecedent of the rules. That is, for Rule (1), 17.9 % of the Adults in the predictive data were covered. The fifth row shows the percentage of records covered by a rule that had Malaria. That is, for Rule (1), when the model was compared with actual classifications in the Test data, it correctly predicted 85 % cases of adults. The results in the preceding columns hold for their age groups and rules respectively.

## Discussion

The prototype relied on two sources of information in order to guide exploration; approximate model using information from Training data set and a utility-model using test data which is used to refine the approximate model’s predictions (Van Bever et al. 2013; Wesonga 2015). The prototype was able to achieve its intended objectives. The system contained an inbuilt database of malaria predictors as well as graphical user interfaces for the user to interact with the predictors.

The sensitivity and specificity tests of goodness of fit and entropy for the rule-based classifiers were employed by the system to test the reliability of the classification rules to diagnose malaria. The goodness of fit statistics show that overall, the data mining classification rules correctly specified 70 % of patients with malaria. A similar trend was noted with specificity that showed a better performance for the adults aged 18 years and above of 72 % while the lowest specificity value was 50 % occurring for both categories of 5–9 and 10–17 year olds respectively. Overall, the sensitivity values for the training dataset (89 %) were higher than those for the predictive dataset (58 %), as well as the specificity values. This is mainly because in deriving the classification rules, data from training dataset (Kalisizo hospital) were used.

On the other hand, the entropy tests of goodness of fit show that the prototype had over 85 % reliability for adult patients aged at least 18 years, which corroborates well with the sensitivity and specificity tests.

The ID3 algorithm adopted for the classification rules prototype is known to ignore irrelevant attributes and only takes into consideration attributes that are relevant to the diagnosis (malaria) problem. This implies that the user is spared the burden of getting spurious results thus improving on efficiency because the algorithm only selects attributes that minimize entropy; implying that the less one is uncertain about the results, the more the information gain and vice versa. On the other hand, there are a number of significant challenges that must be solved to enable the real-world use the prototype. These challenges include: the predictive dataset was predominantly composed of adults since this was the age group with access to Internet so as to access the system. The prototype excluded persons below the age of 5 years because they rarely explicitly express their complaints to the medical worker.

Incorporating real time feedback into the prototype was tricky since potential patients had to return to the system after the confirmatory tests were done. There was a very thin line between Malaria and Typhoid as both have some overlapping clinical features (Cunha 2007). According to (Pradhan 2011) malaria begins with multiple shaking chills, whereas typhoid fever begins with a single morning shaking chill.

## Conclusions

With such statistical systems, data mining classification rules, the cost of malaria treatment, especially in developing countries, could be minimised so as to reduce, in the worst case, some otherwise avoidable mortalities due to malaria. Some system specific conclusions are drawn; firstly, the system comprised of a database of malaria predictors as well as custom driven interfaces to enable users’ interact with the predictors. Secondly, the entropy, sensitivity and specificity tests for the goodness of fit were integrated as predictive models amenable to clinical diagnosis of malaria. Thirdly, rule-based classification algorithm, data mining technique, was used in the architectural design of the system to facilitate malaria diagnosis. Above all the system was tested and found to be more reliable for persons aged 18 years and above.

In summary, the study developed a prototype for predicting the patient’s malarial status. The statistical prototype was evaluated for efficacy showing a sensitivity value of 70 % across age groups. We are cognisant of the fact that any predictive model is not meant to replace clinical diagnosis, rather it provides screening that may require a confirmatory test. However, when appropriately used and integrated into the medical malarial investigatory system, the predictive system’s malarial outcome may provide additional useful information. Lastly, although the system did not take into considerations persons below the age of 5 years, findings based on adults indicate that the system can as well be extended and adjusted for application on the children.
